# Acute Non-ST-Elevation Myocardial Infarction in a 26-Year-Old Woman: A Case Report

**DOI:** 10.7759/cureus.93433

**Published:** 2025-09-28

**Authors:** Munish Sharma, Subina Sapkota, Salim Surani, Anjali M Gaalla, Neha Chandna

**Affiliations:** 1 Pulmonary and Critical Care, Baylor Scott and White Medical Center - Temple, Temple, USA; 2 Pediatrics, Civil Service Hospital, Kathmandu, NPL; 3 Anesthesiology, Mayo Clinic, Rochester, USA; 4 Medicine, Texas A&M University, College Station, USA; 5 Medicine, University of North Texas, Denton, USA; 6 Internal Medicine, Pulmonary Associates of Corpus Christi, Corpus Christi, USA; 7 Clinical Medicine, University of Houston, Houston, USA; 8 Research, Victoria Heart and Vascular Center, Victoria, USA

**Keywords:** acute coronary syndrome, diabetes mellitus, dyslipidemia, early cardiovascular risk screening, hypertension, non-st-elevation myocardial infraction, percutaneous coronary intervention, premature coronary syndrome, young female

## Abstract

Acute coronary syndrome (ACS) in young females is uncommon. The presence of different comorbidities like hypertension, diabetes, and dyslipidemia increases the risk of ACS. Adverse outcomes can be prevented by having early recognition and intervention. A 26-year-old female with a history of hypertension, diabetes mellitus, and dyslipidemia presented with initially intermittent left-sided chest pain radiating to the jaw but progressive in the last five days and associated with left arm numbness. Her ECG showed anteroseptal T-wave inversion, and her troponin level was also elevated. Echocardiography revealed anteroseptal hypokinesia with an ejection fraction of 45%. The diagnosis was made of non-ST-elevation myocardial infarction. Additionally, on coronary angiography, stenosis of the proximal to mid-left anterior descending (LAD) artery was shown, for which she underwent successful percutaneous transluminal coronary angioplasty and drug-eluting stent of the proximal LAD artery. This case shows the importance of early cardiovascular risk screening and intervention, even in the absence of traditional risk factors like smoking. Furthermore, it shows an increasing incidence of premature coronary artery disease in young females with a history of comorbidities like hypertension, diabetes mellitus, and dyslipidemia.

## Introduction

Acute coronary syndrome (ACS) consists of ST-elevation myocardial infarction, non-ST-elevation myocardial infarction, and unstable angina. Among these, non-ST-elevation myocardial infarction (NSTEMI) remains a significant cause of morbidity and mortality worldwide. However, its presentation in young females, especially under 30 years of age, is relatively rare and often underrecognized [1]. Previously, it was considered a condition predominantly affecting males. However, recent studies suggest an alarming increase in cardiovascular events in young women due to metabolic syndrome components such as hypertension, diabetes, and dyslipidemia [1]. In this case, we present a 26-year-old female with no history of smoking or alcohol use who developed NSTEMI but with a history of hypertension, dyslipidemia, and diabetes mellitus, and required urgent percutaneous coronary intervention (PCI) for LAD artery stenosis.

According to the WHO, cardiovascular diseases are the cause of most non-communicable disease (NCD) deaths. About 19 million deaths in 2021. Metabolic changes that increase the risk of NCDs include high blood pressure, obesity, overweight, diabetes, and abnormal blood lipids. Among these, the leading metabolic risk factor globally is elevated blood pressure; about 25% of global NCD deaths are attributed to this, followed by raised blood glucose and overweight/obesity. Among the cardiovascular diseases, coronary artery disease (CAD) is the most common cause. In the USA, about one in 20 adults aged 20 and older has CAD. In 2023, about one out of every six deaths from cardiovascular diseases was in adults younger than 65 years old [2-3].

## Case presentation

A 26-year-old female presented with symptoms of left-sided chest pain on and off, with left arm numbness. According to her, the chest discomfort started three days ago. She developed acute chest pain while dancing, which radiated to the right side of her jaw. Initially, she thought it was a tooth problem, but the pain did not subside, and she was taken to the emergency room and was later found to have elevated troponin. On admission, the patient seemed to be comfortable. Her blood pressure was 133/90 mmHg, and her heart rate was 106 beats per minute. On review of the cardiovascular system, she had chest pain with radiation to the jaw and left arm, and left arm numbness in the neurological system; however, the systemic examination was unremarkable.

Her past history was significant for hypertension, diabetes on insulin glargine, and hyperlipidemia on statin, with no history of smoking or alcohol consumption. In the emergency room, she was started on a heparin drip using a weight-based protocol, an aspirin and Plavix loading dose, and nitroglycerin 0.4 mg sublingual as needed.

Investigations

The ECG showed a normal sinus rhythm with some anteroseptal T-wave inversion. Additionally, echocardiography revealed a mild decrease in left ventricular systolic function with anteroseptal hypokinesia and 45% of ejection fraction, mild mitral and tricuspid insufficiency, and mild left atrial dilation. Her troponin level was 2684 nanograms/liter (normal range less than 4 ng/l), creatinine kinase-MB 6.1 nanograms/liter (0-3.9 ng/ml), and creatinine kinase was around 100 U/L (MOU2) (25 to 200 U/L). The detailed lab data are shown in Table 1.

**Table 1:** Baseline laboratory values WBC: white blood cells, RBC: red blood cells, MCV: mean corpuscular volume, MCH: mean corpuscular hemoglobin, MCHC: mean corpuscular hemoglobin concentration, PLT: platelets, BUN: blood urea nitrogen, CO₂: carbon dioxide, GFR: glomerular filtration rate, PT: prothrombin time, INR: international normalized ratio, POC: point of care, CK-MB: creatine kinase–myocardial band

	Lab values	Ranges
WBC	9.9	4.8-10.8 10 x 3/ul
RBC	4.13 L	4.30-6.00 10 x 6/UL
Hemoglobin	8.9 L	11.5-16G/dl
Hematocrit	28.2 L	35-48%
MCV	68.3 L	80-100 fl
MCH	21.6 L	26-34 pg
MCHC	31.6 L	32-36 g/dl
PLT	244	130-450 10 x 3/ul
Sodium	138	135-145 mmol/l
Potassium	3.5	3.5-5.0 mmol/l
Chloride	105	95-112 mmol/l
Glucose, quant	148 H	70-100
Calcium	7.9	8.7-10.7 mg/dl
BUN	16	6.0-20.0 mg/dl
CO2	25	21-31 mmol/l
Creatinine	0.94	0.05-1.20 mg/dl
Anion gap	11.5	8-16
GFR	>60	-
Calculated osmolality	280	270-300 mosm/kg
PT	12.9 H	11.0-12.8 secs
INR	1.1	0.9-1.1
POC glucose whole blood	357 CH	70-100 mg/dl
CK-MB	6.1 CH	0-3.9 ng/ml
Troponin I	2684 NG/L	Below 0.04 ng/l
Pregnancy test	Negative	

**Figure 1:**
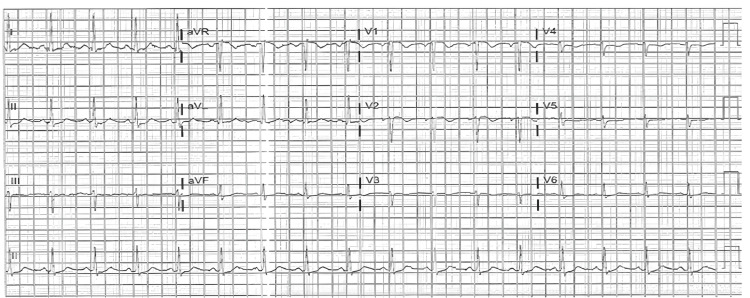
12-lead ECG demonstrating sinus tachycardia with a heart rate of approximately 105 beats per minute. There are non-specific ST-segment and T-wave abnormalities noted, without acute ST-segment elevation or depression. No pathological Q waves or significant arrhythmias are observed. ECG: electrocardiogram

**Figure 2:**
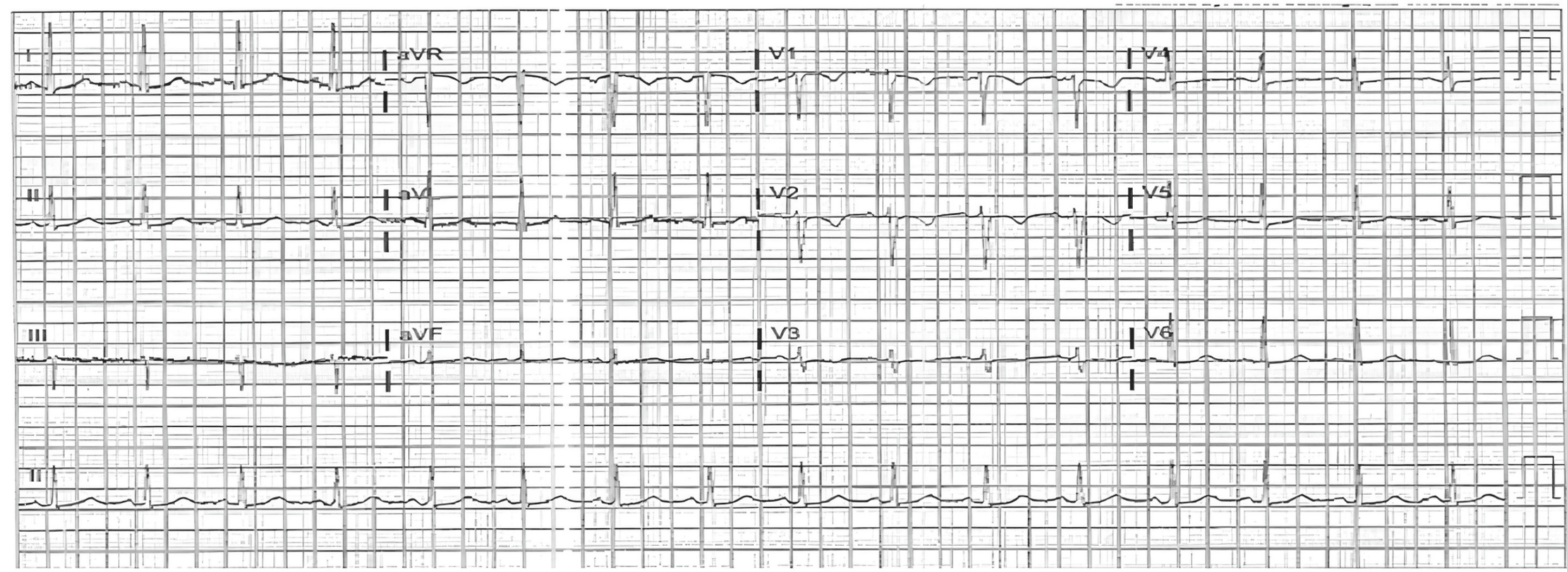
12-lead ECG demonstrating sinus tachycardia with a heart rate of approximately 105 beats per minute. There are non-specific ST-segment and T-wave abnormalities noted, without acute ST-segment elevation or depression. No pathological Q waves or significant arrhythmias are observed. ECG: electrocardiogram

**Figure 3:**
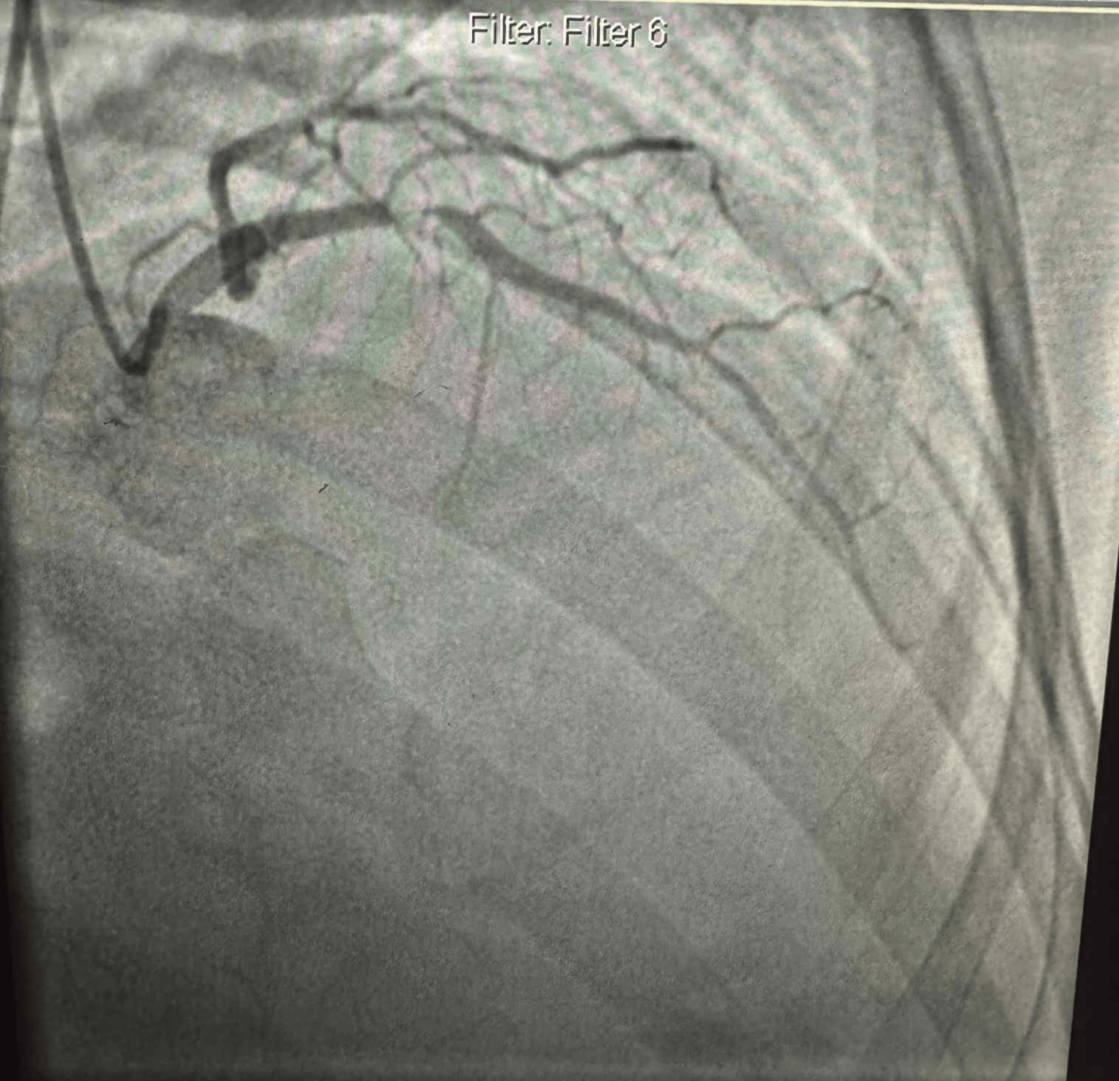
Coronary angiography in the right anterior oblique view revealed a severe mid-LAD stenosis, while the circumflex artery appeared patent. LAD: left anterior descending

**Figure 4:**
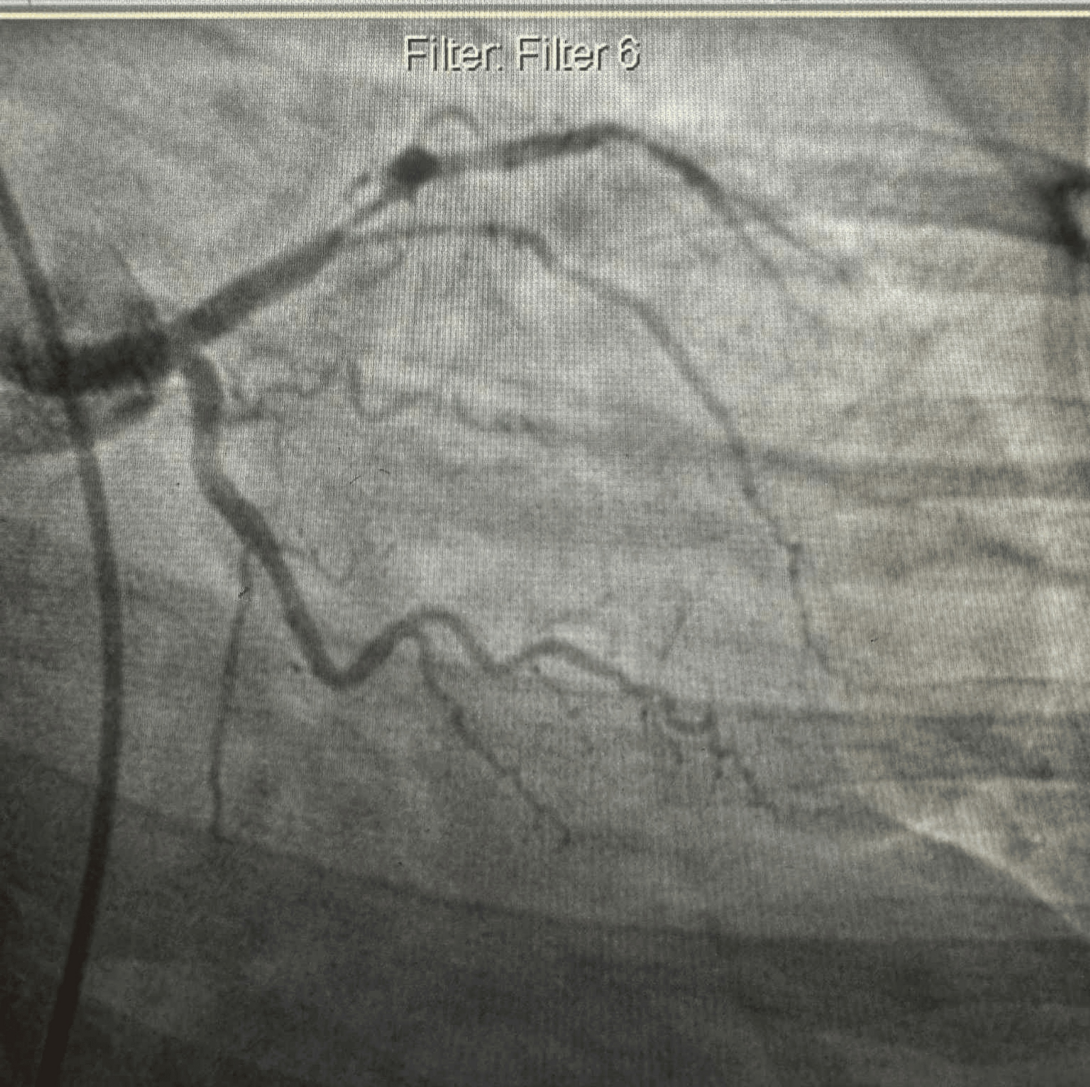
Coronary angiography in the anterior-posterior caudal view revealed a severe mid-LAD stenosis, while the circumflex artery appeared patent.

**Figure 5:**
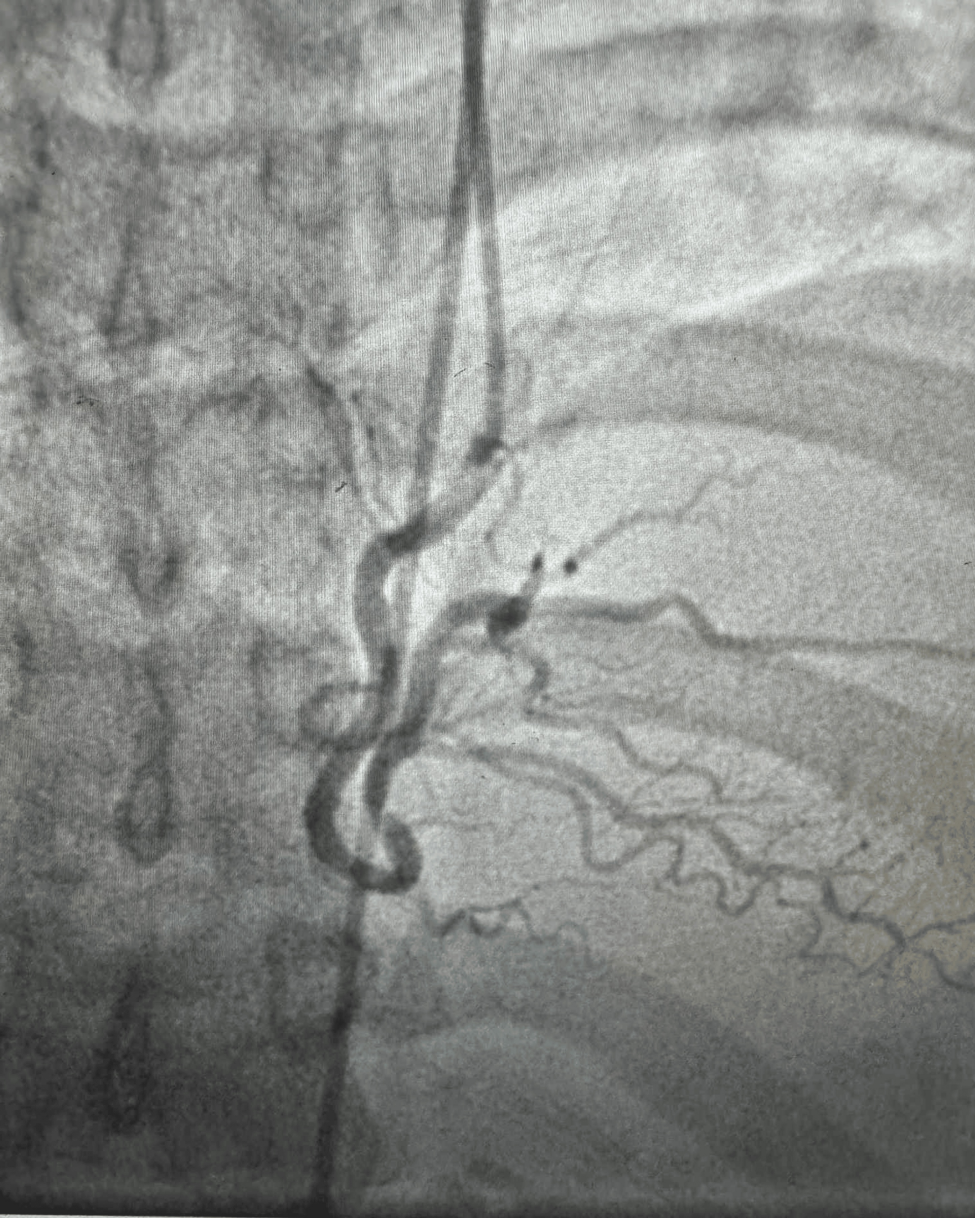
Coronary angiography showed no significant diseases in the right coronary artery.

**Figure 6:**
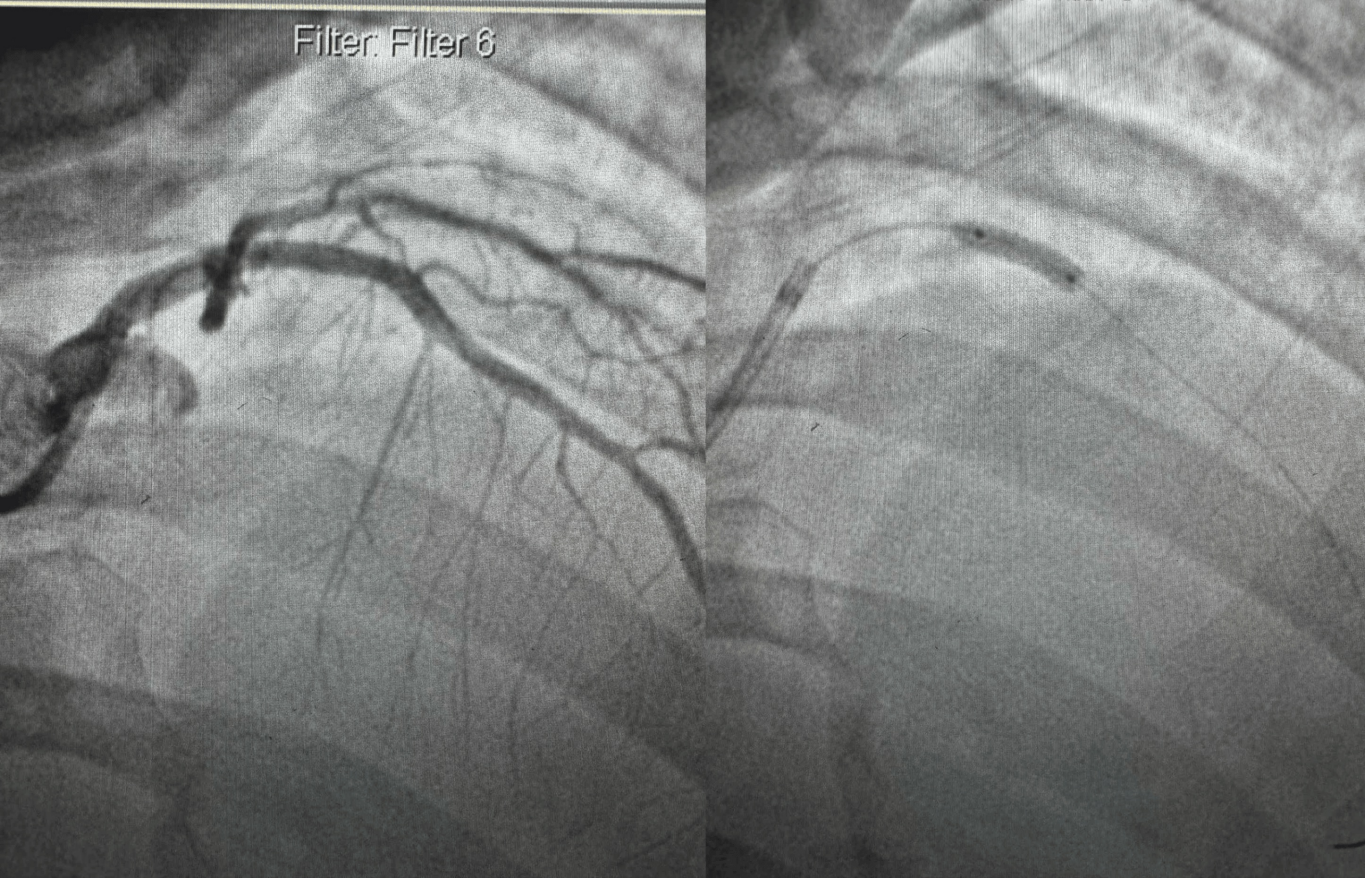
Coronary angiography image in the right anterior oblique view showing balloon angioplasty and deployment of a drug-eluting stent in the proximal to mid-segment of the LAD artery. Post-stenting, the LAD is patent with no residual stenosis. LAD: left anterior descending

With elevated cardiac enzymes, abnormal electrocardiography (Figures 1-2), and an echocardiogram, the diagnosis of ACS (NSTEMI) was confirmed. The patient underwent a coronary angiogram, revealing stenosis of the proximal to mid-left anterior descending (LAD), for which she underwent percutaneous transluminal coronary angioplasty, and a drug-eluting stent of the proximal and mid-LAD was performed (Figures 3-6). The lesion appeared as severe (almost 99%) stenosis in the proximal to mid LAD with thrombolysis in myocardial infarction 3 flow. Intravascular imaging was not performed in this case. However, spontaneous coronary artery dissection (SCAD) was considered part of the differential. There were no typical features of SCAD, such as multiple radiolucent lumens, contrast staining, or long smooth narrowing without atherosclerotic changes.

The patient's hospitalization course was stable, and she was discharged home within 48 hours on a low-dose aspirin regimen (81 mg), clopidogrel (75 mg) daily, and atorvastatin (40 mg) daily, in addition to her home insulin regimen, as mentioned before. The patient was stable and doing well at her outpatient follow-up visits, with the latest visit occurring six months post-procedure.

## Discussion

This case shows an evolving pattern of premature CAD in young women. Here, our patient had major metabolic risk factors: hypertension, diabetes mellitus, and dyslipidemia. These factors synergistically contribute to endothelial dysfunction, atherosclerosis, and plaque instability. Despite her young age, the presence of these comorbidities placed her at significant risk.

ACS in a 26-year-old female is often not recognized since it is typically common in the middle-aged or older population due to the increasing obesity, diabetes, and chronic kidney disease, and the increasing detection of myocardial necrosis by troponin [4]. Therefore, it is important to maintain a high index of suspicion for myocardial infarction even in young patients, especially those with multiple comorbidities.

This patient had strong risk factors, including hypertension, diabetes mellitus, and dyslipidemia. These likely contribute to accelerated atherosclerosis and early CAD. Her presentation with elevated troponin and non-ST changes on ECG led to the diagnosis of NSTEMI, and left heart catheterization, selective coronary angio, and left ventricular angiogram findings suggest CAD with critical stenosis of the proximal to mid-LAD artery. Additionally, the patient underwent percutaneous transluminal coronary angioplasty and a drug-eluting stent of the proximal LAD artery and was discharged in stable condition.

From the study of risk factors of ACS in young age, risk factors like diabetes mellitus, high BMI, obesity, hypercholesterolemia, hypertension, smoking, and family history increased the acute coronary risk in young women. A total of 7,042 patients met the inclusion criteria for this meta-analysis, as reported in seven studies [5]. Screening methods, such as routine blood pressure measurement, fasting lipid profile, hemoglobin A1c, or fasting blood glucose testing, along with assessment of BMI/waist circumference, can aid in early diagnosis in young adults with a family history or multiple risk factors.

Additionally, from the study of Modifiable Risk Factors in Young Adults With First Myocardial Infarction, 1,462,168 young adults with a first acute myocardial infarction (mean age 50 +-7 years, 71.5% men, 58.3% White), of whom 19.2% were 18 to 44 years of age. In the 18- to 44-year-old group, smoking (56.8%), dyslipidemia (51.7%), and hypertension (49.8%) were most prevalent, and 90.3% of patients had at least one risk factor. Women had a higher prevalence of diabetes mellitus, hypertension, and obesity, and men had a higher prevalence of dyslipidemia, drug abuse, and smoking [6]. SCAD, anomalous origin or course of coronary artery, coronary vasospasm, stress-induced cardiomyopathy, myocarditis, and myopericarditis are some of the cardiac differential diagnoses that should be kept in mind in a young patient presenting with ACS-like symptoms.

According to the 2025 American College of Cardiology/American Heart Association/American College of Emergency Physicians/National Association of Emergency Medical Service Physicians/Society for Cardiovascular Angiography Intervention guideline for the management of patients with ACS, our patient underwent PTCI targeting the LAD [7]. This patient was at intermediate to high risk of ischemic event and was an appropriate candidate for revascularization, an invasive approach with intent to proceed with revascularization recommended during hospitalization to reduce MACE [7]. Early diagnosis and aggressive secondary prevention are essential to reduce future cardiovascular events and mortality.

## Conclusions

This case highlights the importance of recognizing premature atherosclerosis, particularly in patients with multiple risk factors. Additionally, a high index of suspicion for ACS in young women, especially those with multiple cardiovascular risk factors, should be maintained. It reinforces the fact that traditional age and gender stereotypes should not guide clinical judgment. It supports early cardiovascular screening, risk factor control, and timely intervention to reduce the global burden. Early diagnosis through appropriate imaging and biomarkers, timely PCI, and aggressive risk factor modification remain the cornerstone of management. Secondary prevention can also be done by taking statins, ACE inhibitors, beta blockers, antiplatelets, and strict lifestyle modification for long-term prognosis.
